# Does a home treatment acute relapse prevention strategy reduce admissions for people with mania in bipolar affective disorder?

**DOI:** 10.1192/pb.bp.113.044321

**Published:** 2014-12

**Authors:** Claudia Murton, Michael Cooper, Stephen Dinniss, Shon Roberts, Nicola Booth, Paul Newell

**Affiliations:** 1 Plymouth Community Healthcare; 2 Plymouth University

## Abstract

**Aims and method** To assess whether a home treatment team acute relapse prevention (ARP) strategy reduces admissions to hospital with mania. A retrospective design was used to analyse records for manic admissions since 2002. The number and length of admissions and detentions pre- and post-ARP were determined and rates of admissions and detentions calculated from this.

**Results** We found reductions in admission and detention rates following the introduction of the ARP: 0.3 fewer admissions per person per year (95% bootstrap CI 0.09–0.62) and 0.25 fewer detentions per person per year (95% bootstrap CI 0.0–0.48). Wilcoxon signed-rank tests gave *P*<0.0001.

**Clinical implications** A person-centred care plan such as the ARP which enables quick action in response to relapse-warning signs of mania appears to reduce rates of admission to hospital. The ARP could be used anywhere in the UK and fits with current mental health policy.

Bipolar affective disorder is relatively common, with a lifetime prevalence of approximately 1.3%.^1^ Mental illness such as bipolar affective disorder causes significant morbidity.^2^ It is a severe mood disorder and people who experience relapses, particularly of mania, are at increased risk of being admitted to hospital.^3^

The acute relapse prevention (ARP) strategy is a person-centred collaborative care plan for people with bipolar affective disorder who are known to mental health services in Plymouth and is used specifically in a manic phase. It was designed by members of the home treatment team (HTT) in Plymouth in 2007, following concerns that service users with early relapse signs were not felt to reach HTT thresholds and therefore not taken on to case-load. This led to rapid deterioration and subsequent admissions to psychiatric hospital (either voluntary or compulsory admissions under the Mental Health Act 1983 – detentions) which were felt to have been potentially avoidable if quicker action with targeted treatment had been taken.

Each person with bipolar affective disorder known to the service is given an opportunity to create their own personalised version of the care plan in collaboration with professionals from the HTT and carers or family if desired. The plan identifies specific relapse signatures for individuals and interventions which can help prevent further deterioration. With the person’s consent the plan is shared with professionals involved in their care and allows general practitioners (GPs), community teams, families, carers or the individual themself to refer to the HTT and be instantly taken on to case-load with no initial assessment, to expedite immediate initiation of their personalised treatment plan. The ARP aims to intervene as early as possible in mania. The purpose of this is to reduce further deterioration, which can be rapid, minimise disruption to lives and help avoid hospital admissions and detentions. People are taken on by the HTT for an initial period of 4 days for assessment and treatment if appropriate. Subsequently, they either stay with the HTT for a longer period or are transferred to their care coordinator or general practitioner.

Personalised, collaborative care plans are part of mental health policy in the UK;^2^ however, there has been little work done to quantify their use in allowing instant access to an HTT when early relapse signs of mania are detected which may otherwise fall below the threshold for action by a home treatment or crisis team. No literature was identified which evaluated a structured acute relapse prevention strategy, such as that in Plymouth, in an HTT. However, there is evidence promoting the benefit of helping people with bipolar affective disorder recognise signs of relapse to control their condition and reduce its impact on their lives.^4–8^

The study was undertaken to evaluate this specific care plan by finding out whether voluntary and involuntary admissions to hospital were reduced for this group of people after initiation of their ARP care plan.

## Method

In total, 59 people were included in the analysis. They became known to the service between 4 March 2002 and 25 October 2010 and were aged 18 years or over. The ARP care plans were implemented during the period 19 March 2007 to 21 February 2011. The number of days that people were known to the service prior to the start of an ARP care plan ranged from 28 to 1841, and 435–1870 days since the start of their ARP care plan.

Electronic records for service users were retrospectively analysed from 2002 until data collection in May 2012. Depressive relapses were excluded from the data by reading electronic records.

Data were used to identify the number of admissions to psychiatric hospital people had before and after an ARP was put into place for them, and the total number of days spent in hospital pre- and post-ARP. They were also used to identify the number of detentions pre- and post-ARP and the total days spent detained pre- and post-ARP. People were either detained under Section 2 (a compulsory admission to hospital for assessment in hospital for up to 28 days) or Section 3 (compulsory admission for treatment for up to 6 months) of the Mental Health Act.

Rates of admissions and detentions pre- and post-ARP were calculated for each person (admissions/detentions per person per year) to allow for differing time periods before and after an ARP was introduced.

## Statistical analysis

Statistical analysis was carried out using the ‘R’ environment for statistical computing with the ‘lattice’ package for producing graphics.^9,10^ Data were checked for distributional assumptions using QQ-plots.

For each patient the difference between the pre-ARP and post-ARP rates was calculated, which allows for the different time periods that each person was known to the service pre- and post-ARP. We then tested whether the median of the differences was equal to 0 using Wilcoxon signed-rank tests: *P*<0.05 was determined to be statistically significant; 95% confidence intervals for the median

**Table 1:** Summary statistics for ward admissions and detentions under the Mental Health Act

	Pre-ARP	Post-ARP
Ward admissions		
Total, *n*	105	54
Patients admitted, *n*	46	21
Length of admission, days: median (IQR)	26 (12-47)	26 (8-53.75)
Patient admission rate^a^: median (IQR)	0.51 (0.22-1.33)	0.0 (0.00-0.45)
Difference in admission rate^a^: median (95% CI)	0.3 (–0.62 to –0.09)****	
		
Detentions under the Mental Health Act		
Total, *n*	96	38
People detained, *n*	39	15
Length of detention, days: median (IQR)	26 (4.5-36)	23 (1.25-66.75)
Patient detention rate^b^: median (IQR)	0.40 (0.00-1.14)	0.0 (0.00-0.10)
Difference in detention rate^b^: median (95% CI)	0.25 (–0.48 to 0.00)****	

ARP, acute relapse prevention strategy.

a. Admissions per person per year.

b. Detentions per person per year.

**** *P*<0.0001 (calculated from Wilcoxon signed-rank tests of person-specific differences in admission rates; 95% CIs are estimated from percentiles of bootstrap samples of the median difference in admission rates)

differences were calculated using the percentiles from a bootstrap sample.

## Results

### Ward admissions

Of the 59 people, 46 (80%) were admitted at least once pre-ARP, with 105 admissions in total, and 21 people (36%) were admitted at least once post-ARP, with 54 admissions in total (Table 1). The number of admissions for both groups ranged from 1 to 9 per person; 11 people (19%) were never admitted with mania.

The number of admissions per year pre-ARP ranged from 0.2 to 12.6 and post-ARP from 0.2 to 1.8. The total number of days spent admitted to hospital was 4162 pre-ARP and 2102 post-ARP.

#### Annual admission rates

**Fig 1:**
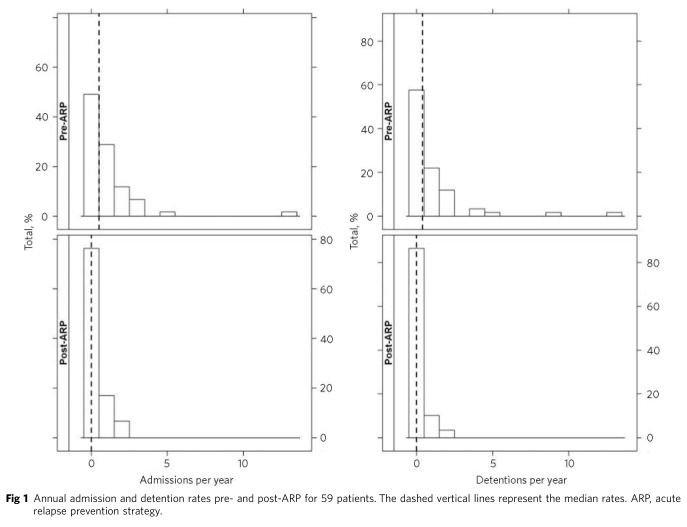
Annual admission and detention rates pre- and post-ARP for 59 patients. The dashed vertical lines represent the median rates. ARP, acute relapse prevention strategy.

The median patient admission rates were 0.51 admissions per person per year pre-ARP and 0.0 per person per year post-ARP, as illustrated in Fig. 1. The median difference in admission rates was –0.3 per person per year, equating to almost 1 less admission over 3 years (95% CI –0.62 to –0.09). A Wilcoxon signed-rank test on the differences in admission rates gave *P*<0.0001. Therefore, there is evidence that the median difference in admission rates per person per year pre- and post-ARP is not 0.

### Detentions under the Mental Health Act

Overall, 39 people in the study (66%) were detained at least once pre-ARP, with 96 detentions in total and 1 to 8 detentions per person. Post-ARP, 15 people (25%) were detained at least once (range 1 to 8 detentions) and there were 38 detentions in total. There were 42 Section 2 detentions pre-ARP and 10 post-ARP; and 37 Section 3 detentions pre-ARP and 16 post-ARP; 20 people (34%) were never detained with mania.

The number of detentions per year pre-ARP ranged from 0.2 to 12.6 and post ARP from 0.2 to 2.3. The total number of days spent detained was 2976 pre-ARP and 1947 post-ARP.

#### Annual detention rates

The median patient detention rates were 0.4 detentions per person per year pre-ARP and 0.0 per person per year post-ARP, as illustrated in Fig. 1. The median difference in detention rates was –0.25 per person per year, equating to around 1 less detention over 4 years (95% CI –0.48 to 0.00).

A Wilcoxon signed-rank test on the differences in detention rates gives *P*<0.0001. However, the bootstrap confidence interval for the median difference in detention rates includes 0. Therefore, there is some evidence that the median difference in detention rates per person per year pre- and post-ARP is not 0.

## Discussion

There were fewer admissions and detentions post-ARP than pre-ARP via the HTT, and statistical testing of the differences in the rates of admissions and detentions revealed statistically significant reductions in the rates of admissions and detentions post-ARP compared with pre-ARP. There were 0.3 fewer admissions per person per year equating to almost 1 less admission over 3 years and 0.25 fewer detentions per person per year equating to approximately 1 less detention over 4 years. Although the bootstrap confidence interval for the median difference in detention rates includes 0, there is still some evidence that the median difference in detention rates is not 0. The median length of stay per admission was not significantly reduced.

These may seem relatively small values, but given the amount of disruption that can be caused to a person’s life when they become manic, including the risk of being detained in hospital, avoiding even 1 detention every 4 years can be important.

To put our results into context, it has been found that crisis teams such as the HTT can reduce in-patient admissions for people with all diagnoses.^11,12^ However, it is also known that people with severe mood disorders may present particular difficulties in home treatment and were the most likely to later require transfer to in-patient care, therefore it was suggested that particular attention be paid to the initial assessment and treatment plan for such patients.^3,13,14^ There is a large body of evidence advocating early recognition and collaborative management of relapse signs of mania, including guidelines from the National Institute for Health and Care Excellence (NICE).^6^ Self-management approaches based on recognition of early warning signs of mania or depression are also popular with patient groups.^7^ A randomised controlled trial on the efficacy of using a health professional to teach patients with bipolar affective disorder to recognise, and seek treatment for, early warning signs of mania found that time to relapse was increased four-fold in the intervention group, as well as other clinically important improvements in functioning, including employment.^8^ A Cochrane review^5^ found beneficial effects of early warning sign interventions (defined as psychological treatments aimed at helping patients recognise early warning signs themselves) in time to recurrence, percentage of people hospitalised and functioning of people with bipolar affective disorder. Other systematic reviews^15–18^ found psychosocial interventions (defined as any non-pharmacological intervention aimed at improving functioning and targeting prevention of further episodes) to be effective as an adjunct to pharmacological therapy in reducing relapse. However, despite the large body of evidence advocating early recognition and collaborative management of relapse signs of mania,^4–8,16–18^ there is little literature about a person-centred care plan such as the ARP allowing instant access to and treatment by an HTT, in some cases via self-referral. Our findings on this intervention indicate that this is an effective, innovative and useful approach which adds something a little different to the current evidence on early interventions for mania.

Our participants are from Plymouth, which is a medium-sized university town. Figures from UK national statistics specifying the demographics of Plymouth^19^ do not suggest major differences to other urban areas in the UK which could mean our results would be unique. Although our sample is relatively small, it could be expected that our results would be generalisable to other parts of the UK should similar care plans be implemented elsewhere. However, intensive treatment at home may work better in urban populations due to the shorter distances needed to travel.

It is possible that by reducing admissions cost savings are made, although no clear conclusion about this could be drawn from the results of this study. It would be important to ensure an adequately funded and staffed HTT so that crisis response and management could be handled promptly and efficiently. A similar plan could be introduced for bipolar depression and other conditions, such as schizophrenia and borderline personality disorder. However, this type of care plan suits mania in bipolar affective disorder because of the speed of progression in mood elevation that can occur.

### Limitations

A control group could not be analysed, as most people with a history of bipolar affective disorder known to the service in Plymouth have an ARP in place.

Fifty-nine is a relatively small sample size; however, this was the total number of people with an ARP in Plymouth. We were therefore unable to make a power calculation prior to starting the study. Larger studies over several centres could be planned further to this study, for example, a cohort study including a control group over several geographical areas, if the ARP is introduced elsewhere.

There has been an increase in experience and staffing in the Plymouth HTT since 2002, which may have contributed to the reduction in admission and detention rates.

We are aware that by calculating patient-specific admission and detention rates we have not allowed for the duration of these events. For example, a person may have had several shorter admissions that do not last as long in total as another person’s one prolonged admission.

### Message

An ARP strategy in an HTT acts to intervene and treat early at home when people with bipolar affective disorder show relapse-warning signs of mania. Rates of admissions and detentions under the Mental Health Act were statistically significantly reduced in people with bipolar affective disorder and an ARP in Plymouth. The ARP is unique in early warning sign interventions in that it is a care plan aimed at immediate acceptance and intervention from an HTT with strategies known to help an individual. This could be used anywhere in the UK.
